# COVID-19 and Other Pleiotropic Actions of Vitamin D: Proceedings from the Fifth International Conference “Vitamin D—Minimum, Maximum, Optimum” under the Auspices of the European Vitamin D Association (EVIDAS)

**DOI:** 10.3390/nu15112530

**Published:** 2023-05-29

**Authors:** Pawel Pludowski

**Affiliations:** Department of Biochemistry, Radioimmunology and Experimental Medicine, The Children’s Memorial Health Institute, 04-730 Warsaw, Poland; pludowski@yahoo.com

Vitamin D deficiency appeared as a worldwide pandemic markedly earlier than the COVID-19 pandemic was announced in global media. Unfortunately, vitamin D deficiency is still considered a pandemic and has been associated with a variety of diseases, including infectious diseases, autoimmune diseases, neurocognitive dysfunction and several deadly cancers. In recent years, a number of recommendations have been published, providing guidelines on how to protect people from vitamin D insufficiency and how to treat deficiency.

The International “Vitamin D- minimum, maximum, optimum” conference, held under the auspices of the European Vitamin D Association (EVIDAS), was a platform providing recent data for scientists and medical doctors to discuss the pleiotropic actions of vitamin D. The fifth edition of this International EVIDAS conference was organized in Warsaw, Poland, in October 2021, and it was attended by scientists and MDs who were exposed to different topics that were later published in a Special Issue of *Nutrients*. One of the main topics was the problem that many diseases, conditions, or drugs per se were shown to influence the anabolic or catabolic pathways of vitamin D metabolism or to increase or decrease the need for vitamin D supplementation. These questions have been answered, at least by Bleizgys [1], Pludowski et al. [2], Pilz et al. [3] and Grant et al. [4].

The paper by Bleizgys [1] provided the basic principles and a brief algorithm of recommendations for prophylactic and therapeutic issues relating to vitamin D deficiency. The paper reviewed the other available guidelines published for a region of Central Europe, discussed the current needs of new recommendations for clinical practice, described the groups at risk of vitamin D deficiency, including COVID-19 cases, and discussed the dosing of vitamin D for the risk groups. Finally, Bleizgys published a practical algorithm focused on vitamin D dosing in relation to the concentration of 25(OH)D [1].

The other recommendations were established for Central and Eastern Europe [2]. Based on the Delphi method, 10 experts, representing 10 different countries in Europe, decided to update the guidelines for a region inhabited by ca. 300 million people. It should be noted that most of the general populations of these countries already exhibited vitamin D deficiency, and the risk groups were even more affected by vitamin D deficiency and COVID-19. The Central and Eastern European recommendations were focused on adults only; however, the clinical aspects were extensively discussed, and the proper dosing (6000 IU/day for 2–3 months) for the treatment of vitamin D deficiency and an algorithm were published again [2]. Interestingly, this paper discussed the meaning of the potential use of calcifediol, i.e., 25(OH)D; however, it was concluded that “at this stage, we continue to recommend vitamin D3 (cholecalciferol)“ due to the fact that “randomized controlled trials (RCTs) data are still missing on the superior benefit of calcifediol versus vitamin D, with reference to hard clinical outcomes” [2].

The questions focused on the reasons leading Bleizgys [1] or European experts [2] to establish local or regional recommendations were answered in reviews by Pilz et al. [3] and Grant et al. [4]. Pilz et al. provided their point of view on the consequences of epidemiological studies that led to the large vitamin D randomized controlled trials (RCTs) designed, conducted and completed in recent years [3]. It was shown that the RCTs were not focused on individuals with an evident vitamin D deficiency. Moreover, some RCTs even allowed moderate vitamin D supplementation in the placebo groups. Consequently, the major message from these RCTs in the mass media informed populations about the lack of significant effects of vitamin D on the risk of developing cancer, cardiovascular events, or mortality [3]. One aspect raised by the authors was safety. It appeared that RCTs with high doses of vitamin D did not demonstrate significant risks of hypercalciuria, hypercalcemia, nephrocalcinosis or kidney stones [3]. The same was highlighted by Grant et al. [4], although from a more general point of view. Grant et al. [4] pointed out the improper dissemination of data indicating the benefits of vitamin D supplementation that exposed the entire population to an increased risk of cardiovascular disease, hypertension, cancer, type 2 diabetes mellitus and COVID-19. The major outcomes provided by Grant et al. were from ecological and observational studies, studies of mechanisms, and Mendelian randomization studies and randomized controlled trials (RCTs). The latter ones were, however, criticized due to the fact that they were focused on the dose of vitamin D rather than on baseline and achieved concentrations of 25(OH)D, with the participation of subjects demonstrating concentrations of 25(OH)D above the population mean, and some of them were permitted additional sources of vitamin D. It was concluded that raising serum 25(OH)D concentrations to optimal concentrations (30–50 ng/mL) will reduce the risks of illness and death, keeping in mind again the message that the risks of hypercalciuria, hypercalcemia, nephrocalcinosis or kidney stones are so minimal that they may be even omitted [4]. Interestingly, Pilz et al. [5] found a case of a pregnant woman who exhibited hypercalcemia due to pathogenic mutations of CYP24A1. These types of mutations were related to the impaired catabolism of vitamin D metabolites. The diagnosis was based on a reduced 24,25-dihydroxyvitamin D to 25-hydroxyvitamin D ratio and was finally confirmed by genetics. In general, the state of pregnancy in the case studied, in combination with a review of another 13 cases, revealed obstetric complications due to hypercalcemia [5]. Therefore, in such cases, the avoidance of vitamin D supplementation appeared to be effective in preventing or reducing the degree of hypercalcemia [5]. Mutations of CYP24A1 are rare but should be considered in pregnant women as well as in the general population.

A dysregulated immune response to the SARS-CoV-2 virus leading to severe COVID-19 was discussed by Thomas D. Thacher [6]. In his review, the author provided insights into differences between observational studies and randomized controlled trials (RCTs). The most powerful data from the observational studies cited by Thacher [6] were from a retrospective analysis of 191,779 individuals that revealed markedly lower COVID-19 cases among those with greater 25(OH)D concentrations [7]. Concerning RCTs, up to the date of Thacher’s publication, a meta-analysis combining the results of three RCTs and two quasi-experimental studies of vitamin D in COVID-19 found no conclusive evidence that vitamin D supplementation reduced ICU admission, invasive ventilation or mortality [8]. However, in an RCT that included oral calcifediol (25(OH)D) rather than cholecalciferol, this molecule, as noted by Thacher [6], significantly reduced the risk of ICU admission in patients with diagnosed COVID-19 (adjusted OR 0.03) [9].

One of the RCTs not discussed by Thacher [6] was a study conducted in Saint Petersburg, Russia [10]. The study by Karonova et al. aimed to investigate the effectiveness of cholecalciferol use on the immune system in COVID-19 cases. A total of 129 patients were randomized into 2 groups: group I (*n* = 56) received 50,000 IU of cholecalciferol on days 1 and 8 of hospitalization, and group II (*n* = 54) did not. The patients exposed to 100,000 IU of vitamin D, despite reaching a median serum 25(OH)D concentration of 22.8 ng/mL after treatment, revealed positive effects on their immune cells and therefore on the course of COVID-19 [10].

An interesting observational study of COVID-19 cases was performed by Smaha et al. [11]. The study focused on an investigation of how ongoing inflammation in COVID-19 affects 25(OH)D concentration. Blood samples were taken upon admission (day 0) and every 24 h for the next four days (day 1–4). The change in 25(OH)D concentration between hospital admission on day 0 and day 4 was 16% (4.8 ng/mL; *p* < 0.0001). On the last day, i.e., day 4, the prevalence of patients showing a reduced 25(OH)D concentration increased by 18% (*p* = 0.018). The authors concluded that the concentration of 25(OH)D decreased significantly during the first two days. After that, no significant change in 25(OH)D concentration was noted; therefore, the still-unanswered question as to whether a low 25(OH)D concentration in COVID-19 relates to a worse prognosis for this disease or only represents a laboratory phenomenon was raised by authors [11].

Populations in the Middle Eastern and North African (MENA) region were shown to suffer from severe vitamin D deficiencies, as documented by the lowest serum 25(OH)D concentrations. Additionally, the highest prevalence of vitamin D deficiency was noted in Arab women. Among these women, vitamin D deficiency was related to a very conservative style of dress and extremely hot weather during most of the year; both factors led to extremely limited sun exposure. The aim of the study by Al Anouti et al. [12] was to evaluate the prevalence of vitamin D deficiency in the group of female migrants residing in the United Arab Emirates (UAE). It appeared that in 550 young women (age 35 year), the prevalence rate of vitamin D deficiency, defined as 25(OH)D ≤ 20 ng/mL, was 67% (95% CI 60–73%), with the highest rate noted in Arab female migrants (87%), followed by South Asians (83%) and Filipinas (15%). Additional analyses focused on associated risk factors indicated low levels of physical activity or obesity as independently associated risk factors for vitamin D deficiency in migrant women residing in the UAE [12].

In the next publication by Stawicki, Abramowicz and colleagues [13], vitamin D deficiency was investigated in in children with juvenile idiopathic arthritis (JIA) who were treated with methotrexate (MTX) and glucocorticoids (GCs). In the investigated group, the median 25(OH)D was 15.0 ng/mL, and vitamin D deficiency was found in 67.2% of the young patients and was independent of sex, disease manifestation, *C*-reactive protein (CRP), the erythrocyte sedimentation rate (ESR), total alkaline phosphatase (ALP) and phosphate (PO4). Higher doses of MTX but not GCs coincided with lower 25(OH)D concentrations (*p* < 0.05). The JIA patients demonstrated problems with their vitamin D status independent of disease activity or inflammatory markers, and authors concluded that vitamin D deficiency should be considered and properly treated in JIA children [13].

A post hoc analysis of the RCT Styrian Vitamin D Hypertension Trial (2011–2014), with 200 hypertensive cases showing 25(OH)D concentrations < 30 ng/mL, was published by Theiler-Schwetz et al. [14]. The authors decided to additionally investigate the effect of cholecalciferol treatment in a daily dose of 2800 IU (or a placebo; 1:1) for 8 weeks on 24 h systolic ambulatory blood pressure (BP), with *p* values < 0.0026 considered significant, keeping in mind the need to correct for multiple testing [14]. Of the 188 patients included, 37% had 25(OH)D < 20 ng/mL, 21% had 25(OH)D < 16 ng/mL and 7% of patients had a 25(OH)D concentration lower than 12 ng/mL. Although the antihypertensive treatment with the use of 2800 IU daily was not effective, a significant trend of an inverse association between the achieved 25(OH)D concentration and systolic pressure was noted (*p* = 0.003).

The controversy surrounding the non-calcemic benefits of vitamin D supplementation led the scientists to perform studies, and the results were presented during the fifth edition of the International “Vitamin D- minimum, maximum, optimum” (EVIDAS) conference. This conference provides a unique forum for basic scientists, clinical researchers, physicians and health care professionals to hear about and discuss these controversies. The next conference, i.e., the sixth edition of the International “Vitamin D- minimum, maximum, optimum” (EVIDAS 2023) conference, which will be held on 22–23 September 2023 in Warsaw, Poland, will provide participants with access to the most recent data on vitamin D and calcifediol. The results will be published in the next Special Issue in *Nutrients,* titled “Pleiotropic Actions of Vitamin D: Proceedings from the 6th International Conference “Vitamin D—Minimum, Maximum, Optimum”, and the information is available at the following link: https://www.mdpi.com/journal/nutrients/special_issues/11EX54YL3Q (accessed on 10 May 2023).

## Data Availability

Not applicable.
